# CYC27 Synthetic Derivative of Bromophenol from Red Alga *Rhodomela confervoides*: Anti-Diabetic Effects of Sensitizing Insulin Signaling Pathways and Modulating RNA Splicing-Associated RBPs

**DOI:** 10.3390/md17010049

**Published:** 2019-01-11

**Authors:** Jiao Luo, Bo Jiang, Chao Li, Xiaoling Jia, Dayong Shi

**Affiliations:** 1 School of Public Health, Qingdao University, Qingdao 266071, China; luojiao2012@163.com; 2 CAS Key Laboratory of Experimental Marine Biology, Institute of Oceanology, Chinese Academy of Sciences, Qingdao 266071, China; jiangbo@qdio.ac.cn (B.J.); lichao161@mails.ucas.edu.cn (C.L.); jiaxiaoling161@163.com (X.J.); 3 State Key Laboratory of Microbial Technology, Shandong University, Jinan 250100, China

**Keywords:** brominated organic compounds, hypoglycemic activity, insulin-sensitizing effect, RBPs, RNA splicing

## Abstract

RNA-binding proteins (RBPs) lie at the center of posttranscriptional regulation and the dysregulation of RBPs contributes to diabetes. Therefore, the modulation of RBPs is anticipated to become a potential therapeutic approach to diabetes. CYC27 is a synthetic derivative of marine bromophenol BDB, which is isolated from red alga *Rhodomela confervoides*. In this study, we found that CYC27 significantly lowered the blood glucose levels of diabetic BKS db mice. Moreover, CYC27 effectively ameliorated dyslipidemia in BKS db mice by reducing their total serum cholesterol (TC) and triglyceride (TG) levels. Furthermore, CYC27 was an insulin-sensitizing agent with increased insulin-stimulated phosphorylation of insulin receptors and relevant downstream factors. Finally, to systemically study the mechanisms of CYC27, label-free quantitative phosphoproteomic analysis was performed to investigate global changes in phosphorylation. Enriched GO annotation showed that most regulated phosphoproteins were related to RNA splicing and RNA processing. Enriched KEGG analysis showed that a spliceosome-associated pathway was the predominant pathway after CYC27 treatment. Protein-protein interaction (PPI) analysis showed that CYC27 modulated the process of mRNA splicing via phosphorylation of the relevant RBPs, including upregulated Cstf3 and Srrt. Our results suggested that CYC27 treatment exerted promising anti-diabetic effects by sensitizing the insulin signaling pathways and modulating RNA splicing-associated RBPs.

## 1. Introduction

Diabetes is a global epidemic. The IDF Diabetes Atlas, eighth edition, shows that, globally, adult diabetes has increased from 151 million cases in 2000 to 425 million in 2017; this is an increase of nearly two times, meaning that one in every 11 adults has diabetes. Type 2 diabetes mellitus (T2DM), which accounts for 90–95% of all diabetes cases, is characterized by insulin resistance and/or impaired insulin secretion, resulting in hyperglycemia [1]. When diet and exercise cannot sufficiently maintain blood glucose levels, proper medical intervention is needed. Currently, the FDA has approved 12 classes of drugs to control the blood glucose levels of T2DM patients, including sulfonylureas, biguanide and α-glucosidase inhibitors [2]. However, these agents each have their limitations and defects. For example, the first-line drug, metformin, which has no well-defined molecular target, can cause gastrointestinal side effects [3]. Other classes of drugs also have some drawbacks, such as weight gain, heart failure and hypoglycemia [4,5,6]. Moreover, glucose control of T2DM patients can become progressively insensitive over time and, subsequently, requires a new drug treatment every 3–4 years for the purpose of maintaining glucose control [7]. Therefore, successful intervention of T2DM needs a continuous supply of new agents.

RNA binding proteins (RBPs) comprise a large and diverse group. They are located at the center of posttranscriptional regulation because they govern RNA biogenesis, export, translation and decay [8,9]. Several RBPs have been reported to be dysregulated in diabetes. LIN28 increases cell glucose uptake and ameliorates insulin resistance in diabetic mice induced with a high-fat diet. Conversely, LIN28 with muscle-specific deletion increases insulin resistance, indicating that LIN28 has an important role in modulating insulin sensitivity [10]. IGF2 mRNA binding protein 2 (IGF2BP2) has been identified as a strong susceptibility gene for T2DM in many human genetic studies [11,12], and a single nucleotide polymorphism (SNP) in its intron is associated with T2DM by genome-wide association studies. Although the correlation between RBPs and T2DM has been determined, cognition of RBPs in skeletal muscle cells is still in the early stages and the functions of most RBPs remain unknown.

**Figure 1:**
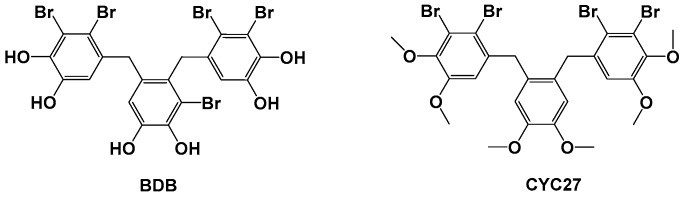
Structures of BDB and CYC27.

Protein tyrosine phosphatase 1B (PTP1B) is a crucial negative regulator of insulin signaling, which directly interacts with insulin receptors (IR) and insulin receptor substrate 1 (IRS1) to dephosphorylate tyrosine residue. PTP1B directly affects several molecular signaling pathways implicated in insulin resistance. Therefore, inhibition of PTP1B is a convincing approach for the treatment of T2DM [13,14]. Our PTP1B small molecule inhibitor program started with the optimization of natural compounds isolated from the marine red alga *Rhodomela confervoides*. The lead compound BDB, 3-bromo-4,5-bis(2,3-dibromo-4,5-dihydroxybenzyl)pyrocatechol, was a kind of bromophenol and was identified as a PTP1B inhibitor with IC_50_ 1.7 μM (structure shown in Figure 1) [15]. Among the synthetic derivatives of BDB, the compound CYC27 showed the most potent PTP1B inhibition (IC_50_ = 0.89 μM, structure shown in Figure 1) [16]. Hence, we chose to investigate the mechanism of action and anti-diabetic efficacy of CYC27 in BKS db mice, an animal model of T2DM. Here, we found that CYC27 had significant hypoglycemic activity in diabetic mice. To systematically investigate the global protein phosphorylation difference, label-free quantitative phosphoproteomic analysis was performed in C2C12 myotubes after CYC27 stimulation. One RBP network related to RNA splicing was identified after bioinformatics analysis.

## 2. Results

### 2.1. Seven-Week Oral Administration of CZC27 Ameliorates Hyperglycemia in Diabetic Mice

**Figure 2:**
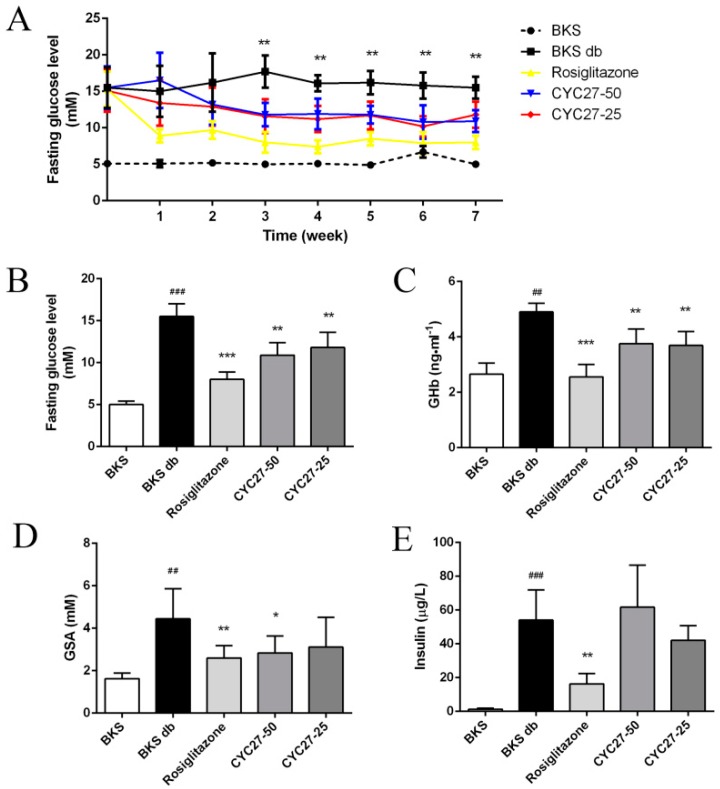
Hypoglycemic effect of CYC27. (**A**) seven week measurement of fasting blood glucose (FBG) levels in BKS db mice after oral administration with vehicle, CYC27 and Rosiglitazone, and in BKS mice treated with vehicle. Data are expressed as mean ± SD (*n* = 8). ** *p* < 0.01, CYC27-50 group versus BKS db group. (**B**–**E**) show the blood glucose levels, GHb levels, GSA levels and insulin levels, respectively, at the seventh week. Data are shown as mean ± SD (*n* = 8). BKS group ^##^ *p* < 0.01 and ^###^ *p* < 0.01 versus BKS db group * *p* < 0.05, ** *p* < 0.01 and *** *p* < 0.001. BKS—vehicle-treated normal BKS mice; BKS db—vehicle-treated diabetic BKS db mice; Rosiglitazone—50 mg·kg^−1^·day^−1^ Rosiglitazone-treated BKS db mice; CYC27-50—50 mg·kg^−1^·day^−1^ CYC27-treated BKS db mice; CYC27-25—25 mg·kg^−1^·day^−1^ CYC27-treated BKS db mice.

To study the anti-diabetic effect of CYC27, fasting blood glucose (FBG) levels were tested once a week. Diabetic BKS db mice had hyperglycemia with blood glucose levels of approximately 15 mM compared with BKS mice (Figure 2A). A seven week administration with CYC27 and Rosiglitazone showed a significant reduction in blood glucose levels compared with the levels of the vehicle-treated diabetic mice (*p* < 0.01, Figure 2A,B). In detail, CYC27 significantly lowered FBG levels from the third week, while Rosiglitazone significantly decreased blood glucose levels from the first week (*p* < 0.01). 

Next, we investigated the effect of CYC27 on glycosylated hemoglobin (GHb) and glycosylated serum albumin (GSA) levels, two well-known parameters of glycemic control. As shown in Figure 2C,D, the serum GHb and GSA levels of BKS db mice significantly increased at the seventh week compared to BKS mice (*p* < 0.01). High-dose CYC27 (50 mg/kg) significantly lowered GHb and GSA levels compared with the vehicle-treated group (*p* < 0.01 and 0.05, respectively, Figure 2C,D). However, low-dose CYC27 (25 mg/kg) significantly reduced the levels of GHb (*p* < 0.01) but had no effect on GSA levels in BKS db mice. Similar to the effect of CYC27, Rosiglitazone also significantly reduced serum GHb and GSA levels in BKS db mice (*p* < 0.01, Figure 2C,D). However, CYC27 had no effect on the serum insulin levels of diabetic mice while Rosiglitazone significantly alleviated hyperinsulinemia (Figure 2E).

### 2.2. CYC27 Ameliorates Dyslipidemia in BKS db Mice

**Figure 3:**
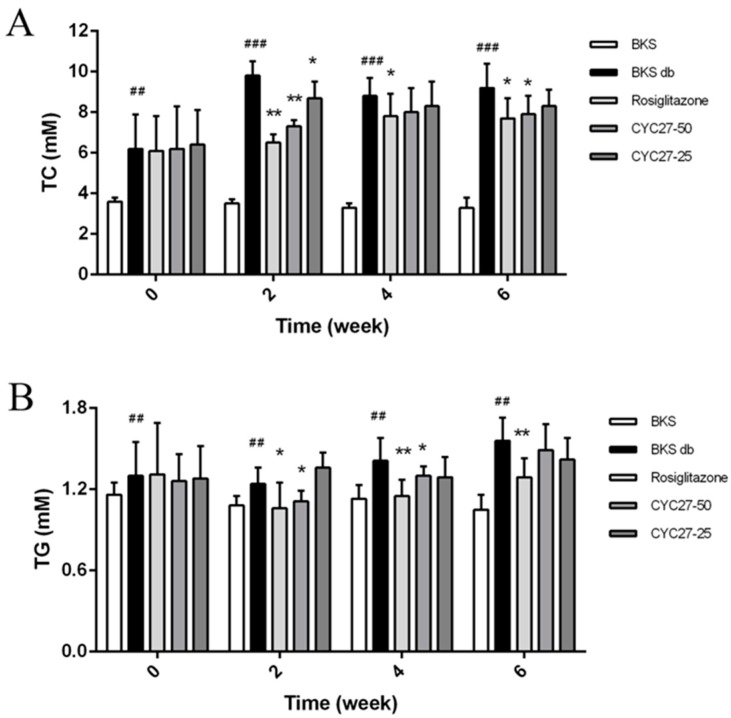
Effects of CYC27 on dyslipidemia. Data in (**A**,**B**) represent the serum total cholesterol (TC) and triglyceride (TG) levels during the seven week administration with the vehicle, CYC27 and Rosiglitazone, and in BKS mice treated with the vehicle. Data are expressed as mean ± SD (*n* = 8). BKS group ^##^ *p* < 0.01 and ^###^ *p* < 0.01 versus BKS db group * *p* < 0.05 and ** *p* < 0.01. BKS—vehicle-treated normal BKS mice; BKS db—vehicle-treated diabetic BKS db mice; Rosiglitazone—Rosiglitazone, 50 mg·kg^−1^·day^−1^ Rosiglitazone-treated BKS db mice; CYC27-50—50 mg·kg^−1^·day^−1^ CYC27-treated BKS db mice; CYC27-25—25 mg·kg^−1^·day^−1^ CYC27-treated BKS db mice.

To further study the effect of CYC27 on dyslipidemia, the serum total cholesterol (TC) and triglyceride (TG) levels were evaluated every two weeks. The results showed that CYC27 significantly lowered serum TC and TG levels beginning in the second week (Figure 3A,B). A more detailed analysis revealed that by the second week CYC27 reduced serum TC levels by 25.5% (*p* < 0.01) and decreased TG levels by 10.5% (*p* < 0.05), compared with vehicle-treated BKS db mice.

### 2.3. No Effect of CYC27 on Bodyweight and Appetite of Diabetic Mice

**Figure 4:**
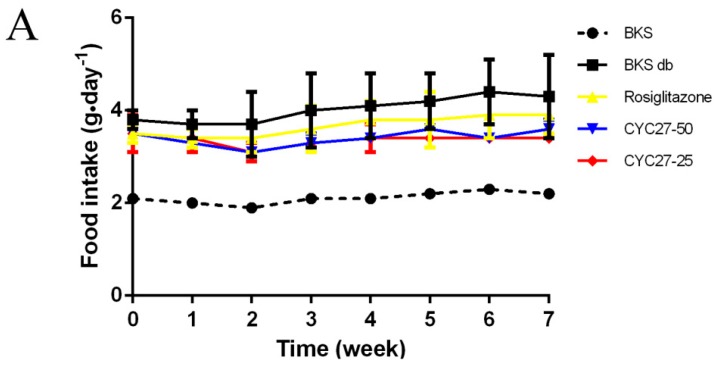

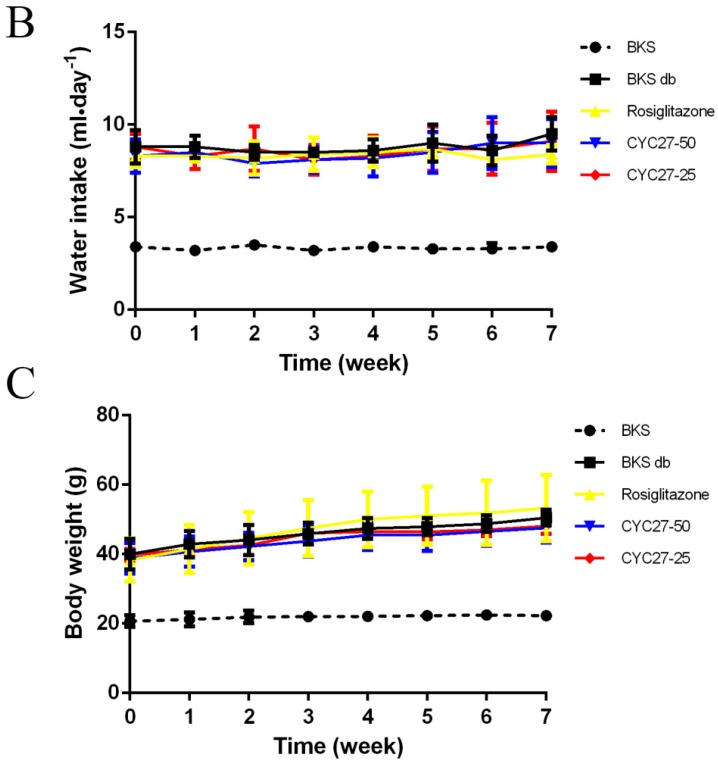
Effects of CYC27 on mean food intake, water intake and body weight during the seven week treatment. (**A**–**C**) show the food intake, water intake and body weight in BKS db mice during the seven week treatment with the vehicle, CYC27 and Rosiglitazone, and in BKS mice treated with vehicle. Data are shown as mean ± SD (*n* = 4). BKS—vehicle-treated normal BKS mice; BKS db—vehicle-treated diabetic BKS db mice; Rosiglitazone—Rosiglitazone, 50 mg·kg^−1^·day^−1^ Rosiglitazone-treated BKS db mice; CYC27-50, 50 mg·kg^−1^·day^−1^ CYC27-treated BKS db mice; CYC27-25—25 mg·kg^−1^·day^−1^ CYC27-treated BKS db mice.

Polydipsia and polyphagia are the characteristic symptoms of T2DM so the effects of CYC27 on bodyweight, food intake and water intake during the seven week treatment were studied. At the beginning of the experiment, there was no difference in bodyweight, food intake and water intake among all groups of diabetic mice (Figure 4A–C). Although there were no statistically significant changes to food intake, the dynamic monitor of CYC27-treated diabetic mice showed that they had a tendency to decrease food intake during the seven week treatment (Figure 4A). As shown in Figure 4B, CYC27 had no effect on the water intake of BKS db mice. Furthermore, diabetic BKS db mice were more obese than normal BKS mice. However, both CYC27 and Rosiglitazone (an insulin sensitizing drug for T2DM) had no effect on the body weight of BKS db mice (Figure 4C).

### 2.4. Insulin-Sensitizing Effect of CYC27 in C2C12 Myotubes 

**Figure 5:**
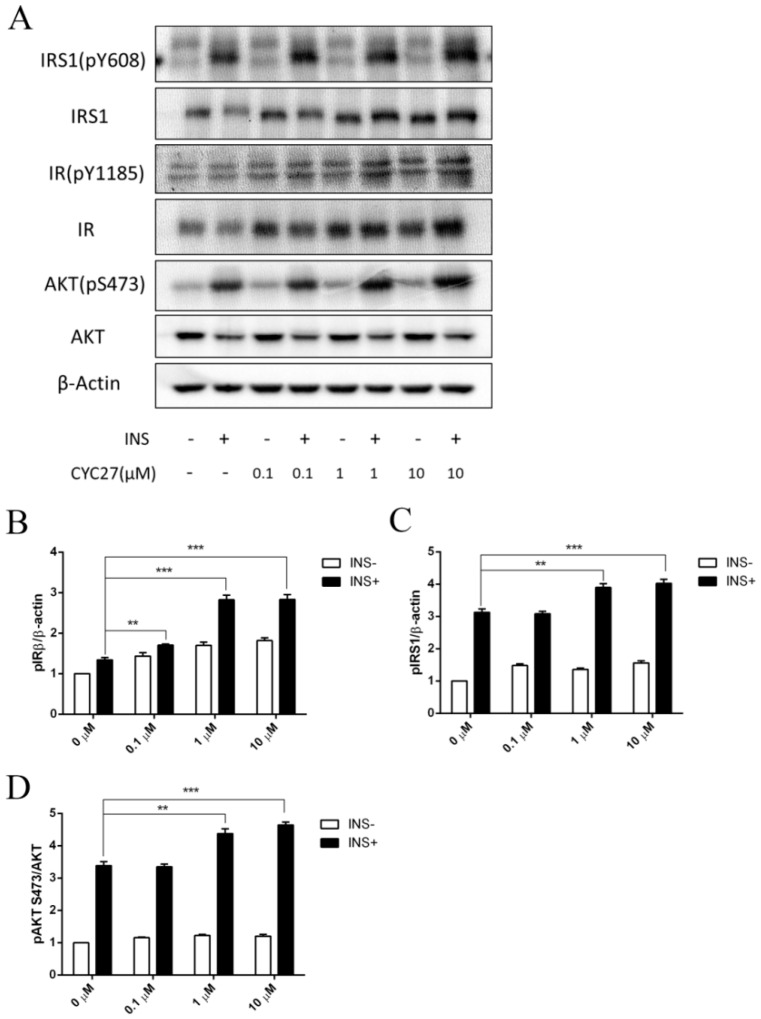
Enhanced insulin signaling by CYC27. (**A**) Effects of CYC27 on insulin signaling transduction. C2C12 myotubes were first starved overnight and then treated with CYC27 for 8 h followed by 100 nM insulin stimulation for 5 min. The phosphorylation level was determined by western blotting in IRβ, IRS1 and Akt. (**B**) Quantification of IRβ phosphorylation. Each band density was quantified and normalized to a β-actin signal. (**C**) Quantification of IRS1 phosphorylation. Each band density was quantified and normalized to a β-actin signal. (**D**) Quantification of Akt phosphorylation. Band density was quantified and then normalized to a total Akt signal. Data are expressed as mean ± SEM (*n* = 3). ** *p* < 0.01 and *** *p* < 0.001 versus non-insulin-treated groups.

Insulin action depends on the action of the insulin receptor (IR) and the downstream signaling factors on the insulin signaling pathway. Tyrosine phosphorylation of the β subunit of the IR and insulin receptor substrate 1 (IRS1) was examined by immunoblotting to determine the effect of CYC27 on insulin signaling. Insulin-stimulated phosphorylation of IRβ (Tyr1185) was enhanced by CYC27 treatment in the C2C12 myotubes (Figure 5A,B). Phosphorylation of IRS1 (Tyr608) was also increased (Figure 5A,C). Consistent with IRβ/IRS1 activation, the insulin-stimulated phosphorylation of downstream factor Akt (Ser473) was also upregulated (Figure 5A,D). Moreover, the activity of CYC27 was dosage-dependent at concentrations between 0.01 and 10 μM (Figure 5B–D). These results suggested that CYC27 improved the activity of insulin signaling in C2C12 skeletal muscle cells.

### 2.5. Label-Free Quantitative Phosphoproteomic Characterization of CYC27-Treated C2C12 Myotubes 

To further investigate the mechanisms of C2C12 myotubes in response to CYC27 treatment, quantitative phosphoproteomic study was used to fully monitor global changes of phosphoproteins. In the present study, a total of 5430 phosphopeptides derived from 2119 proteins with 7363 phosphosites were identified. Significant up/downregulations between samples for phosphopepetide quantification were found based on two criteria: a fold change >2 or <0.5 and *p* value < 0.05. Among the 5430 phosphopeptides, the phosphorylation levels of 195 phosphopeptides were markedly upregulated and 141 phosphopeptides were markedly downregulated. These up/downregulated phosphoproteins were used for a subsequent bioinformatics analysis.

**Figure 6:**
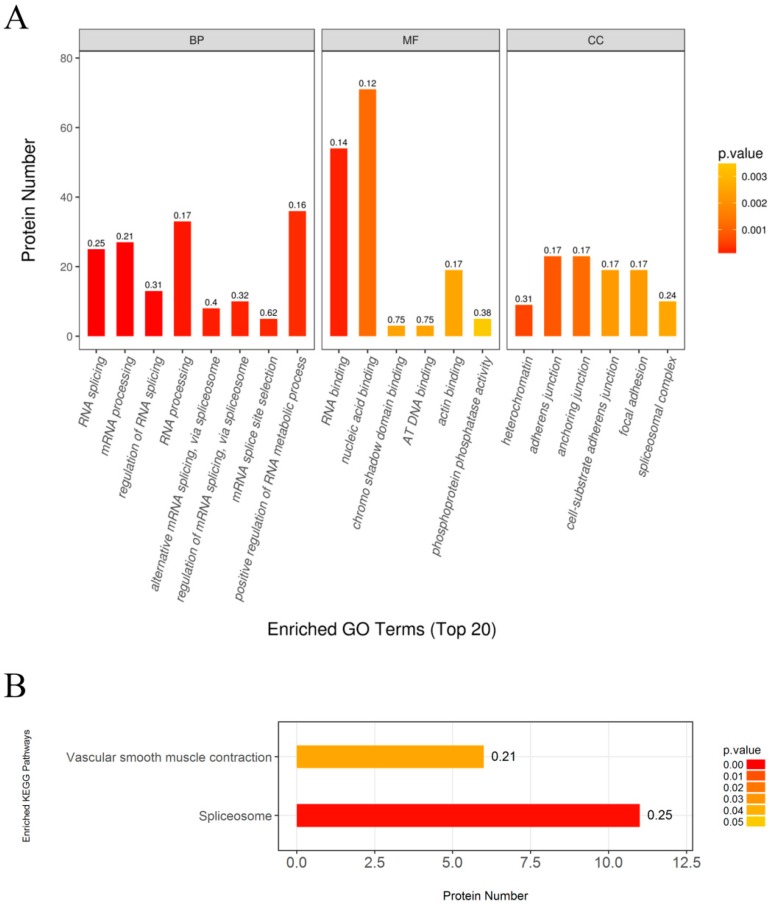
GO terms enrichment and KEGG pathway enrichment of differently phosphorylated proteins in C2C12 myotubes in response to CYC27 treatment. (**A**) GO enrichment analysis. Top 20 enriched GO terms shown. BP—biological progress; MF—molecular function; CC—cellular component. (**B**) Enriched KEGG pathway analysis. *p* < 0.05 considered significant.

First, Gene Ontology (GO) annotation was used to further understand the functions of these identified phosphoproteins, including biological process (BP), molecular function (MF), and cellular component (CC) (Figure 6A). In the biological process categories, a large number of regulated phosphoproteins were related to RNA splicing, mRNA processing, RNA processing and positive regulation of RNA metabolic process. Other regulated phosphoproteins were assigned to the regulation of RNA splicing, alternative mRNA splicing via spliceosome, regulation of mRNA splicing via spliceosome and mRNA splice site selection. 

In the molecular function categories, most regulated phosphoproteins were related to RNA binding and nucleic acid binding. Only a small number of regulated phosphoproteins were assigned to chromo shadow binding, AT DNA binding, actin binding and phosphoprotein phosphatase activity. Among these, RNA binding was the predominant function in response to CYC27 treatment.

In the cellular component categories, the regulated phosphoproteins were divided into six categories, including heterochromatin, adherens junction, anchoring junction, cell-substrate adherens junction, focal adhesion and spliceosome complex. These data indicated that most regulated phosphoproteins originated from adhesion proteins.

Then, KEGG enrichment analysis was performed to evaluate the enrichment of the proteomics pathways. The results indicated that the spliceosome pathway was the predominant pathway associated with CYC27 treatment (Figure 6B).

### 2.6. CYC27 Modulates Phosphorylation of RNA Splicing-Related RBPs 

**Figure 7:**
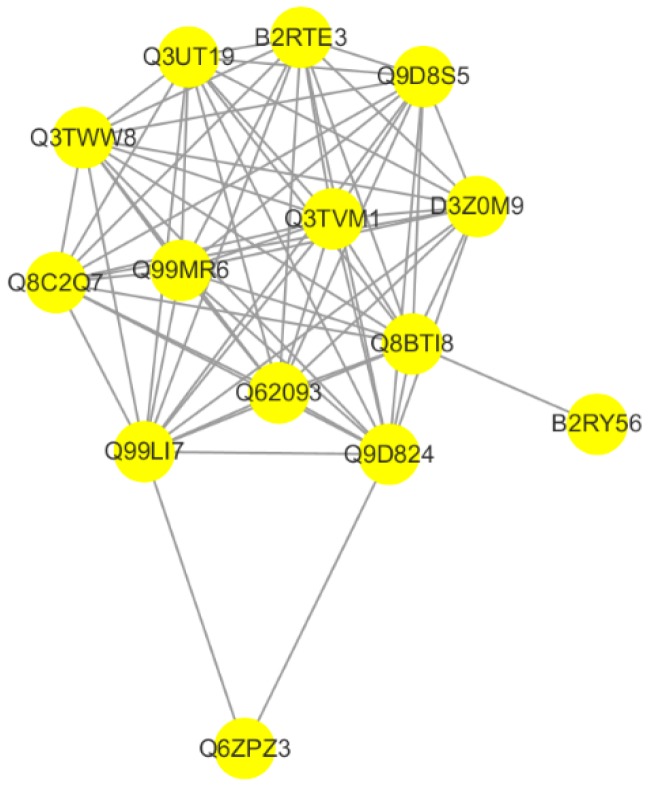
Protein–protein interaction network analysis. Network based on known protein-protein interactions using the STRING database was created and imported the proteomic data into the Cytoscape (version 3.5.1).

Protein–protein interaction (PPI) networks provide a better understanding the functional framework of the identified proteins. Thus, a network based on known protein–protein interactions was created using the STRING database and shown using Cytoscape. Fourteen proteins were included in the network. The protein interaction network showed that the RNA binding protein network, which was involved in the process of RNA splicing, was directly linked to CYC27 treatment in C2C12 myotubes (Figure 7). The detailed difference is shown in Table 1. Among these, the phosphorylation levels of Cstf3, Srrt, Fip1l1, Srsf5, Srrm2, Srsf2 and Rbm25 were significantly upregulated, and the phosphorylation levels of Prpf38a, Ddx23, Prpf3 and Hnrnph1 were significantly downregulated.

## 3. Discussion

Asia is a major area of the rapidly emerging T2DM global epidemic, with China and India as the top two epicenters [17]. The nature of the unmet medical needs for T2DM urges the development of new drug candidates [7]. Here, we described a brominated organic compound, CYC27, which had positive hypoglycemic effects in diabetic mice. In the present study, BKS db mice were used for measuring hypoglycemic effects. BKS db mice are a classic T2DM animal model, which are used to model phases one to three of T2DM and obesity [18]. CYC27 significantly reduced the blood glucose levels of diabetic BKS db mice beginning in the second week of the seven week administration (Figure 2A). The level of plasma GHb was the gold standard for the assessment of glycemic control in patients with type 2 diabetes [19]. In the seventh week, blood GHb and GSA levels were significantly lowered by CYC27, reflecting effective glycemic control of diabetic mice in the past one to two months. More importantly, glycemic control is the basis of T2DM treatment [20]. Our results demonstrated that CYC27 had an anti-diabetic function with effective blood glucose control.

Dyslipidemia is common in diabetic patients with increased TG and TC levels [21]. Moreover, dyslipidemia is a major factor contributing to the accelerated cardiovascular disease in T2DM-associated complications [22]. Management of dyslipidemia in patients with diabetes is needed [23]. In this paper, serum TC and TG levels were significantly reduced by the second week (Figure 3A,B), suggesting the lipid-lowering capability of CYC27 in the BKS db mice. The lowered TC and TG levels in the peripheral circulation might further contribute to the anti-diabetic effects of CYC27.

In the present study, CYC27 had no effect on the bodyweight and appetite of diabetic mice, which means CYC27 was not a weight loss drug. Recently, a novel regulator of insulin resistance Nfe2l1, independent of weight gain, has been described to increase insulin sensitivity of ob/ob mice [24]. Nfe2l1 has great potential to be a therapy target of diabetes and obesity and may be a ligand of many natural and synthetic compounds. 

Insulin signaling is initiated when insulin binds to the extracellular α-subunits of the IR. This binding causes autophosphorylation of several tyrosine residues within the IR β-subunit. The tyrosine phosphorylation of the β-subunit further induces the phosphorylation of the IRS1 at key tyrosine residues, which in turn triggers signaling transduction by activating downstream factors including Akt [25]. Failure of these signals is associated with obesity and the progressive failure of pancreatic beta cells that lead to diabetes [26,27,28]. In the present study, CYC27 improved the activity of the insulin signaling pathways with an increase in insulin-stimulated tyrosine phosphorylation of the IR (Figure 5A). This finding was consistent with studies demonstrating statin-induced activation of the insulin signaling pathway [29]. Our data also showed that CYC27 treatment increased the insulin-stimulated IRS-1/PI3K/Akt pathway, which reinforced previous studies. The insulin-sensitizing effect of CYC27 might have an important role in the improvement of glucose levels and dyslipidemia in diabetic BKS db mice because this pathway had been implicated in glucose transport in muscle and lipid metabolism for liver and adipose tissues [30].

Now what needs to be answered is the crucial question of how CYC27 activates insulin signaling, which in turn carries out the hypoglycemic effect in diabetic mice. To explore the mechanisms of CYC27 activation of the insulin pathway, a label-free quantitative phosphoproteomic technique was used in the present study. In total, 336 differentially expressed phosphopeptides were identified. The phosphorylation levels of 195 phosphopeptides were markedly upregulated, and 141 phosphopeptides were markedly downregulated. Enriched GO annotation showed that most regulated phosphoproteins were related to RNA splicing, mRNA processing and RNA processing (Figure 6A). Enriched KEGG analysis showed that the spliceosome-associated pathway was the predominant pathway after CYC27 treatment (Figure 6B). Research on the network of protein interactions facilitates the in-depth study of molecular mechanisms. A protein–protein interaction (PPI) network was constructed based on the differentially expressed phosphoproteins. PPI analysis showed that CYC27 modulated the process of mRNA splicing via phosphorylating relevant RBPs (Figure 7). RBPs are important regulators of posttranscriptional RNA networks, which are dysregulated in diabetes. Targeting RBPs can be a promising therapy to metabolic disorders [31]. Although important discoveries about possible diabetes-related RBPs were revealed in this paper, there were also limitations. Because there were no commercial antibodies to detect the phosphorylation levels of mRNA splicing-related RBPs, the results of the present label-free LC/MC experiment cannot be validated. 

In summary, as a potent PTP1B inhibitor, CYC27 treatment exerted promising anti-diabetic effects by activating the insulin signaling pathway. RNA splicing-associated RBPs might also participate in the hypoglycemic effect. Overall, these results provided important new insight into the mechanisms of CYC27 action in the treatment of diabetes. 

## 4. Materials and Methods 

### 4.1. Materials

CYC27 was synthesized and identified by our lab according to the previously reported procedure [16] (purity 98%). Rosiglitazone and insulin were purchased from Sigma-Aldrich (St. Louis, MO, USA). DMEM, horse serum and penicillin–streptomycin were obtained from Hyclone (South Logan, UT, USA). FBS was purchased from PAN (Adenbach, Bavaria, Germany). RIPA lysis buffer and PMSF were bought from Solaibo (Beijing, China). Antibodies directed against IRS1, InsRβ and p-Akt (Ser473) were bought from Cell Signaling Technology (Danvers, MA, USA). Phospho-IRβ (Tyr1185) antibody was purchased from Abcam (Cambridge, MA, USA). Antibodies against phospho -IRS1 (Tyr608) and PVDF membrane were bought from Millipore (Bedford, MA, USA). β-actin antibodies and all secondary antibodies were purchased from the Proteintech Group (Wuhan, Hubei, China). The BCA Protein Assay Kit was bought from Beyotime Biotechnology (Shanghai, China). Clarity^TM^ Western ECL Substrate was purchased from Bio-Rad (Hercules, CA, USA). The GHb, GSA, TC and TG assay kit were bought from Nanjing Jiancheng Bioengineering Institute (Nanjing, Jiangsu, China).

### 4.2. Animal studies

Male BKS db mice (BKS.Cg-Dock7^m+/+^Lepr^db^/J, 6–8 weeks, the Jackson Lab stock number 000642) and lean BKS mice (C57BLKS/J, 6–8 weeks, the Jackson Lab stock number 000662) were purchased from the Model Animal Research Centre of Nanjing University (MARC). Mice were housed in isolated ventilated cages in a specific pathogen free (SPF) room and fed with a normal chow diet. All animal procedures were conducted in accordance with the appropriate regulatory standards under the protocol HAIFAJIZI-2013-3 (approval date: 2013-12-09) approved by the Animal Care and Use Committee of Institute of Oceanology, Chinese Academy of Sciences.

After one week of adaptation, all diabetic BKS db mice were randomly divided into four groups (*n* = 8): diabetic model group (BKS db), Rosiglitazone-treated group (Rosiglitazone, 50 mg·kg^−1^·day^−1^), CYC27 high-dose group (CYC27-50, 50 mg·kg^−1^·day^−1^) and CYC27 low-dose group (CYC27-25, 25 mg·kg^−1^·day^−1^). Age-matched male BKS mice were used as the lean wild type control (BKS, *n* = 8). Mice were starved overnight and then tail blood was assessed to measure fasting glucose levels using an Accu-Chek Performa glucometer (Roche, Germany). To measure the serum TG and TC levels, blood samples were collected from the orbital venous plexus every two weeks. TG and TC levels were estimated according to the manufacturers’ introduction from Nanjing Jiancheng Bioengineering Institute. After a seven week administration, mice were fasted overnight and the blood samples were collected to measure the blood GHb and GSA levels. GHb and GSA levels were tested according to the manufacturers’ introduction (Nanjing Jiancheng Bioengineering Institute, China).

### 4.3. Cell Culture and Immunoblotting

C2C12 myocytes were cultured in a high glucose DMEM medium supplemented with 10% FBS. In detail, 4 × 10^5^ C2C12 cells/well were plated in a 6-well plate, and 10% horse serum was added to induce myotube differentiation when the cell density reached 90%. After a four day differentiation, myotubes were starved overnight with a serum-free medium.

Then, C2C12 myotubes were treated with a series dose of CYC27 (0, 0.1, 1 and 10 μM, respectively) for 8 h followed by insulin (100 nM) stimulation or nothing for 5 min. After compound treatment, cells were lysed with pre-cold RIPA buffer. Proteins were separated by gel electrophoresis and then transferred to a PVDF membrane. After, blocked in TBST containing 5% non-fat milk for 1 h at room temperature, membranes were incubated with a primary antibody overnight at 4 °C. The following primary antibodies were used: IRβ, IRβ (pY1185), IRS1, IRS1 (pY608), Akt, Akt (pS473), and β-actin. After overnight incubation, membranes were incubated with a HRP-conjugated secondary antibody for another 1 h. Bands were detected using Bio-Rad Clarity^TM^ Western ECL Substrate.

### 4.4. Label-Free Quazntitative Phosphor-Proteomic Assay 

#### 4.4.1. Protein Extraction, Digestion, and Phosphopeptide Enrichment

Differentiated C2C12 myotubes were treated with DMSO (Control group) or 1 μM CYC27 (CYC27 group) for 8 h. Both groups were stimulated with 100 nM insulin for 5 min. Biological triplicates were mixed for the following test. Cells were lysed with an SDT lysis buffer (4% SDS, 100 mM Tris-HCl, 1 mM DTT, pH 7.6) and homogenized by sonication (80 W, on 10 s, off 15 s, 10 cycles). After centrifugation at 14,000 g for 15 min at 4 °C, the concentration of the supernatant was determined by BCA assay. 

Next, 100–150 μg proteins per sample were washed twice with 200 μL UA buffer (8 M Urea, 50 mM Tris-HCl, pH 8.0) in an ultrafiltration tube (MWCO 10,000, Millipore) by centrifugation at 14,000 g for 15 min. Then, proteins were alkylated by incubation with a 100μL IAA buffer (50 mM IAA in UA) for 30 min in the dark. Subsequently, samples were recovered by centrifugation at 14,000 g for 10 min using an ultrafiltration tube and washed twice with 100 μL UA buffer and then twice with an NH_4_HCO_3_ buffer (50 mM). Finally, samples were digested with 40 μL Trypsin buffer (2–5 μg Trypsin in 40 μL NH_4_HCO_3_ buffer) overnight at 37 °C.

Samples were recovered using the ultrafiltration tube. Then, samples were lyophilized and dissolved in 1 × DHB buffer. TiO_2_ were used to enrich the phosphopeptides, as previously described [32]. Finally, phosphorylated peptide samples were collected and concentrated in vacuo, and dissolved in 25 μL 0.1% FA/H_2_O for the subsequent MS analysis.

#### 4.4.2. LC-MS/MS Analysis

Phosphorylated peptide samples were injected onto a reverse phase trap column (Thermo Scientific) connected with a C18-reversed phase analytical column (Thermo Scientific). The mobile phase buffers were Buffer A (0.1% Formic acid) and Buffer B (84% acetonitrile and 0.1% Formic acid) with a gradient of: 0–35% mobile Phase B from 0–50 min, 35–100% mobile Phase B from 50–55 min, and 100% mobile Phase B from 55–60 min. 

The LC-MS/MS experiment was conducted on a Q Exactive mass spectrometer (Thermo Scientific) coupled with Easy nLC (Thermo Fisher Scientific). Positive ion mode was selected. MS data were acquired using the data-dependent top 10 method. The most abundant precursor ions from the survey scan (300–1800 m/z) were dynamically selected for HCD fragmentation. The AGC target was set to 3e6 and the maximum injection time was set to 10 ms. Forty seconds was selected as the dynamic exclusion duration. A measurement scan was obtained at m/z 200 with a resolution of 70,000, and the resolution of the HCD spectrum was set to 17,500 at m/z 200, and the isolation width was set to 2 m/z. 30 eV was set as the normalized collision energy. The underfill rate was stated as 0.1%. The instrument was run in peptide recognition mode.

#### 4.4.3. Data Analysis

The original MS file was analyzed using MaxQuant software (version 1.5.3.17) [33]. Then, the integrated proteome database uniprot_mouse_83914_20171109.fasta was searched, 6 and 20 ppm, respectively, were chosen as the initial precursor and fragment mass tolerances. During database searches, Carbamidomethyl was a fixed modification; oxidation, acetyl and serine, threonine and tyrosine phosphorylation were variable modifications. We chose up to 2 missed trypsin cleavages. For peptide and protein identification, FDR was set to 0.01 and the minimum peptide length was set to 6. The consensus peptides identified between proteins were combined and shown as one group [34]. Phosphorylated sites were confirmed based on the PTM scores [35]. Potentially phosphorylated sites were defined as highly confident if *p* ≥ 0.75. To determine the differentially expressed phosphopeptides between the control and the CYC27 treated samples, label-free quantification was performed with a minimum of a two-fold change. 

#### 4.4.4. Bioinformatics

For Gene Ontology (GO) annotation, protein sequences of differentially changed proteins were retrieved from the UniProtKB database (Release 2016.10) in FASTA format. A local search of the retrieved sequences against the Swiss Prot database (mouse) using NCBI BLAST to find homologous sequences that could transfer functional annotations to the study sequence. In the present study, the first 10 explosion hits for each query sequence with an E value less than le-3 were retrieved and loaded into Blast2Go (version 3.3.5, BioBam Bioinformatics, Valencia, Spain, https://www.blast2go.com) for GO mapping and annotation [36]. And the E value filter with 1 × 10^−6^, the default progressive EC weight, a GO weight of 5, and the annotation configuration with a comment cutoff of 75 were selected. Then, we re-annotated the uncommented sequence with more relaxed parameters. Sequences without BLAST hits and unannotated sequences were then selected by Inter ProScan [37] for the EBI database to retrieve functional annotations for protein motifs and to incorporate Inter ProScan GO terms into the annotation sets. The GO comment results were drawn by the R script.

For KEGG pathway annotation, sequences of differentially expressed proteins in FASTA format were searched from the online database KEGG (http://geneontologyorg). Retrieved KOs were then mapped to KEGG [38]. 

For the functional enrichment analysis, the intrinsic relationship between differentially expressed proteins was found and the enrichment analysis was conducted. Based on Fisher’s exact test, GO enrichment of biological processes, molecular functions and cellular components, and KEGG pathway enrichment was applied to the entire quantitative protein annotation, which is a background dataset. A *p*-value below 0.05 was considered significant.

Protein–protein interaction (PPI) analysis was performed on mouse protein sequences from the STRING database (http://string-db.org) using BLAST. The network was built based on these interactions and then shown by Cytoscape (version 3.5.1, Institute for Systems Biology, Seattle, WA, USA) software [39].

### 4.5. Statistical Analysis

Data are shown in the format of mean ± SD values. Differences between these mice groups were analyzed by a Student’s t test or one-way ANOVA. A value of *p* < 0.05 was considered significant. SPSS software was used for the statistical analysis.

## Figures and Tables

**Table 1:** Differentially expressed phosphoproteins of RNA splicing-related RBPs.

Unipro ID	STRING Protein	CYC27/Control	Significance A	Function
Q99LI7	Cstf3	7.472584183	0.000326819	One of the multiple factors required for polyadenylation and 3′-end cleavage of mammalian pre-mRNAs
Q99MR6	Srrt	5.784229218	0.00186497	Acts as a mediator between the cap-binding complex (CBC) and the primary microRNAs (miRNAs) processing machinery during cell proliferation
Q9D824	Fip1l1	3.665144836	0.0243222	Plays a key role in pre-mRNA 3′-end formation
Q9D8S5	Srsf5	3.263048411	0.0420283	May play a regulatory role in pre-mRNA splicing
Q8BTI8	Srrm2	4.254634311	0.0113168	Involved in pre-mRNA splicing
Q62093	Srsf2	4.36111003	0.00990027	Necessary for the splicing of pre-mRNA
B2RY56	Rbm25	4.467739807	0.00867081	RNA-binding protein that acts as a regulator of alternative pre-mRNA splicing
B2RTE3	Prpf38a	0.258735802	0.01083	May be required for pre-mRNA splicing
D3Z0M9	Ddx23	0.288561474	0.0184227	ATP-dependent RNA helicase activity in RNA splicing
Q3UT19	Prpf3	0.092103249	0.000013149	Participates in pre-mRNA splicing.
Q8C2Q7	Hnrnph1	0.14592886	0.000383192	Mediates pre-mRNA alternative splicing regulation.
Q3TWW8	Srsf6	0.669694785	0.377717	Plays a role in constitutive splicing and modulates the selection of alternative splice sites.
